# Main Chain–Type Block Copolymers through Atom Transfer Radical Polymerization from Double-Decker–Shaped Polyhedral Oligomeric Silsesquioxane Hybrids

**DOI:** 10.3390/polym12020465

**Published:** 2020-02-17

**Authors:** Wei-Cheng Chen, Yu-Hsuan Tsao, Chih-Feng Wang, Chih-Feng Huang, Lizong Dai, Tao Chen, Shiao-Wei Kuo

**Affiliations:** 1 Department of Materials and Optoelectronic Science, Center of Crystal Research, National Sun Yat-Sen University, Kaohsiung 80424, Taiwan; chwei566@gmail.com (W.-C.C.); b043100042@student.nsysu.edu.tw (Y.-H.T.); 2 Advanced Membrane Materials Research Center, Graduate Institute of Applied Science and Technology, National Taiwan University of Science and Technology, Taipei 10607, Taiwan; cfwang@mail.ntust.edu.tw; 3 Department of Chemical Engineering, National Chung Hsing University, 145 Xingda Road, Taichung 402-27, Taiwan; huangcf@dragon.nchu.edu.tw; 4 Fujian Provincial Key Laboratory of Fire Retardant Materials, College of Materials, Xiamen University, Xiamen 361005, China; lzdai@xmu.edu.cn; 5 Ningbo Institute of Material Technology and Engineering, Chinese Academy of Science, Zhongguan West Road 1219, Ningbo 315201, China; tao.chen@nimte.ac.cn; 6 Department of Medicinal and Applied Chemistry, Kaohsiung Medical University, Kaohsiung 807, Taiwan

**Keywords:** POSS, block copolymer, self-assembly, hydrogen bonding

## Abstract

In this study, we synthesized two main chain–type block copolymers featuring hydrogen bond donor and acceptor segments through atom transfer radical polymerization (ATRP) using a bifunctionalized polyhedral oligomeric silsesquioxane (POSS) nanoparticle as the initiator. Hydrosilylation of vinylbenzyl chloride at the two corners of a double-decker silsesquioxane (DDSQ) provided the bifunctionalized benzyl chloride initiator VBC-DDSQ-VBC, which we applied as a platform to prepare a main chain–type polystyrene homopolymer (PS-DDSQ-PS), the diblock copolymer poly(styrene–*b*–4-vinylpyridine) (P4VP-*b*-PS-DDSQ-PS-*b*-P4VP), and the diblock copolymer poly(styrene–*b*–*tert*-butoxystyrene) (PtBuOS-*b*-PS-DDSQ-PS-*b*-PtBuOS) through sequential ATRP. Selective hydrolysis of the *tert*-butoxyl units of PtBuOS-*b*-PS-DDSQ-PS-*b*-PtBuOS yielded the strongly hydrogen bonding diblock copolymer poly (styrene-*b*-vinylphenol) (PVPh-*b*-PS-DDSQ-PS-*b*-PVPh). We used Fourier transfer infrared spectroscopy, nuclear magnetic resonance spectroscopy, size exclusion chromatography, differential scanning calorimetry, mass-analyzed laser desorption ionization mass spectrometry, and transmission electron microscopy to investigate the chemical structures, thermal behavior, and self-assembled nanostructures formed by these main chain–type block copolymers based on DDSQ.

## 1. Introduction

The self-assembled nanostructures (e.g., cylindrical, lamellar, spherical, and double-gyroid structures) that form from immiscible diblock copolymers (BCPs) have received much attention because of their potential applications in, for example, nano-patterning, drug delivery, and photonic crystals [1,2,3,4,5]. The blending of a homopolymer or a BCP into another BCP matrix, to mediate the volume fraction of the BCP segment, is a relatively facile means of obtaining various self-assembled nanostructures [6,7,8,9]. Positioning hydrogen bond donor or acceptor units into the block segments can alleviate macrophase separation in diblock copolymer mixtures; indeed, many experimental and theoretical studies have been undertaken into the preparation of such block copolymer/homopolymer and block copolymer mixtures [10,11,12,13,14,15,16,17,18].

The incorporation of inorganic nanoparticles into self-assembled BCPs in the form of block copolymer/nanoparticle (BCP/NP) composites, with the blends stabilized through specific interactions, has potential applications in nanodevices, photonics, and sensors [19,20,21,22,23]. Polyhedral oligomeric silsesquioxanes (POSSs) are particularly useful nanoparticles for the preparation of hybrid materials, because they typically have cage-like structures with dimensions of 1–3 nm [24,25,26,27,28,29]. In general, functionalized POSS NPs have been prepared with one, two, or eight functionalized units; we have discussed their effects on the self-assembled structures of BCP hybrid complexes in several previous reports [30,31,32]. For example, the number of phenolic functionalities of a POSS NP strongly affects the self-assembled structures formed when blended with poly (styrene–*b*–4-vinylpyridine) (PS-*b*-P4VP) BCPs, stabilized through hydrogen bonding [32]. Furthermore, we have also observed order–order morphological transitions upon increasing the concentrations of functionalized POSS NPs capable of hydrogen bonding [32,33].

In addition to BCP/POSS composites formed through specific physical interactions, covalent chemical bonding of BCPs with POSS NPs has also attracted much interest [34,35,36,37,38,39]. For example, POSS NPs have been positioned at chain ends (e.g., in POSS-*b*-PS [34,35,36], POSS-*b*-PMMA [37,38], and POSS-*b*-PBLG [39,40]) and at junction points (e.g., in PS-*b*-POSS-*b*-PDMS [41]) through living polymerization and controlled living radical polymerization to obtain related hybrid block copolymers. Furthermore, star block copolymers prepared through living polymerization and controlled living radical polymerization, using POSS NPs as cores, have also been widely investigated in the forms of PS-*b*-P4VP [42], PS-*b*-PVPh [42], PS-*b*-PMMA [43], and PEO-*b*-PBLA [44] hybrid block copolymers. To the best of our knowledge, however, main chain–type BCPs based on bifunctionalized POSS NPs have never been reported previously; thus, we became interested in the synthesis of main chain BCPs featuring POSS NPs at their junction points, prepared using nano-sized bifunctional initiators.

**Scheme 1:**
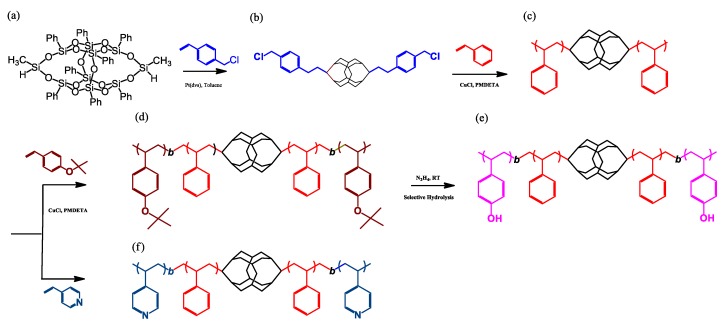
Synthesis of main chain–type block copolymers (**e**) PVPh-*b*-PS-DDSQ-PS-*b*-PVPh and (**f**) P4VP-*b*-PS-DDSQ-PS-*b*-P4VP from (**a**) DDSQ, (**b**) VBC-DDSQ-VBC, (**c**) PS-DDSQ-PS, and (**d**) PtBuOS-*b*-PS-DDSQ-PS-*b*-PtBuOS through selective hydrolysis of (**e**).

In this study, we performed atom transfer radical polymerization (ATRP) of styrene, 4-butoxystryene, and 4-vinylpyridine monomers from a bis (4-vinylbenzyl chloride) double-decker silsesquioxane (VBC-DDSQ-VBC) as the bifunctionalized initiator. We used this approach to synthesize a main chain–type PS homopolymer and the main chain–type diblock copolymers PS-*b*-PVPh and PS-*b*-P4VP, featuring hydrogen bond donor and acceptor units, respectively (Scheme 1). Taking into account the fact that the initiators of VBC-DDSQ-VBC for ATRP were located at the junction points of the DDSQ, the propagation through ATRP extension from such a DDSQ cube appears to be a promising and feasible method for the ATRP-based preparation of well-defined structures. We used Fourier transfer infrared (FTIR) spectroscopy, nuclear magnetic resonance (NMR) spectroscopy, size exclusion chromatography (SEC), differential scanning calorimetry (DSC), mass-analyzed laser desorption ionization time-of-flight (MALDI-TOF) mass spectrometry, and transmission electron microscopy (TEM) to investigate the chemical structures, thermal behavior, and self-assembled nanostructures of these materials.

## 2. Materials and Methods

### 2.1. Materials

Styrene, 4-vinylbenzyl chloride (VBC), 4-*tert*-butoxystyrene, and 4-vinylpyridine were purchased from Aldrich, dried over CaH_2_ and distilled prior to use. Phenyltrimethoxysilane [Scheme 1a], methyl dichlorosilane, tetrahydrofuran (THF), 2-propanol, charcoal, sodium hydroxide (NaOH), ethanol (EtOH), methanol (MeOH), sodium bicarbonate (NaHCO_3_), acetonitrile, cyclohexane, and magnesium sulfate (MgSO_4_) were also purchased from Aldrich. DDSQ was synthesized as described previously [Scheme 1c] [45,46,47].

### 2.2. Bis(4-Vinylbenzyl chloride) Double-Decker Silsesquioxane (VBC-DDSQ-VBC)

A mixture of DDSQ (5 g), toluene (5 mL), VBC (1.33 mL) and Pt (dvs) (0.01 mL) were added and then the mixture was heated at 75 °C with stirring for 48 h to complete hydrosilylation. The mixture was filtered through charcoal and then the filtrate was concentrated through vacuum distillation. The residue was dissolved in CH_2_Cl_2_ and extracted three times with a H_2_O/MeOH mixture. After drying (MgSO_4_, stirring for 30 min), the mixture was filtered to give a clear filtrate, which was dried in a vacuum oven for 24 h at 60 °C to give a white solid [Scheme 1d] (yield: 82%).

### 2.3. PS-DDSQ-PS

A mixture of VBC-DDSQ-VBC (0.380 g), CuBr (0.108 g), and THF (5 mL) was placed into a flask equipped with a reflux condenser and vacuumed by liquid N_2_ three times to deplete water. Styrene (12.0 mL) and PMDETA (0.162 mL) were added and then the mixture was stirred at 110 °C for 6 h. The solution was evaporated through vacuum filtration where alumina oxide was packed to filter Cu ions. The resulting clear solution was concentrated through vacuum distillation and re-precipitated from MeOH to obtain a white filamentous material. The mixture was evaporated through vacuum filtration to obtain a white powder, which was dried in a vacuum oven for 24 h at 60 °C to provide PS-DDSQ-PS as a white solid [Scheme 1e].

### 2.4. P4VP-b-PS-DDSQ-PS-b-P4VP

The mixture of PS-DDSQ-PS (0.5 g) and CuBr (0.0195 g) was placed in a flask equipped with a reflux condenser and vacuumed by liquid N_2_ three times to deplete water. 4-Vinylpyridine (8 mL) and PMDETA (0.03 mL) were added and then the mixture was stirred at 110 °C for 8 and 12 h, respectively. The solution was evaporated through vacuum filtration where alumina oxide was packed to filter Cu ions. The resulting clear solution was concentrated through vacuum distillation and re-precipitated in hexane to obtain a filamentous material. The mixture was evaporated through vacuum filtration to obtain a powder, which was dried in a vacuum oven for 24 h at 60 °C to give P4VP-*b*-PS-DDSQ-PS-*b*-P4VP.

### 2.5. PtBuOS-b-PS-DDSQ-PS-b-PtBuOS and PVPh-b-PS-DDSQ-PS-b-PVPh

The mixture of PS-DDSQ-PS (0.25 g) and CuBr (0.01 g) was placed in a flask equipped with a reflux condenser and vacuumed by liquid N_2_ three times to deplete water. 4-*tert*-Butoxystyrene (6.0 mL) and PMDETA (0.015 mL) were added and the mixture vacuumed by liquid N_2_ again. After stirring at 110 °C for 24 h, the solution was evaporated through vacuum filtration where alumina oxide was packed to filter Cu ions. The resulting clear solution was evaporated through vacuum distillation and re-precipitated from MeOH to obtain a filamentous material. The mixture was evaporated through vacuum filtration to obtain a powder, which was dried in a vacuum oven at 60 °C for 24 h, providing the block copolymer PtBuOS-*b*-PS-DDSQ-PS-*b*-PtBuOS as a solid. The block copolymer PVPh-*b*-PS-DDSQ-PS-*b*-PVPh was prepared through the hydrolysis of PtBuOS-*b*-PS-DDSQ-PS-*b*-PtBuOS in 1,4-dioxane under reflux at 85 °C in the presence of HCl (37 wt%) for 24 h. The mixture was neutralized with NaOH solution (5 wt%) to pH 6–7 and then subjected to three dissolve/precipitate cycles [using THF and MeOH/H_2_O solvent mixtures (1:1, *v*/*v*)]. The resulting solid was dried in a vacuum oven overnight at 60 °C.

### 2.6. Characterization

FTIR spectra were recorded at room temperature at a resolution of 4 cm^−1^ within the range from 4000 to 400 cm^−1^ from 32 scans, using a Nicolet 320 FTIR spectrometer and the typical KBr pellet method. NMR spectra were recorded using an INOVA 500 instrument with CDCl_3_ or DMSO-*d*_6_ as the solvent. The molecular weights of the DDSQ-based PS block copolymers were evaluated from their ^1^H NMR spectra; their polydispersity indices (PDIs) were determined using GPC (Waters 510 apparatus) where the molecular weight calibration was used by PS standard and MALDI-TOF mass spectrometry (Bruker Daltonics Autoflex MALDI-TOF mass spectrometer). The thermal properties of the DDSQ-based block copolymers were determined through DSC using a TA Q-20 instrument; the sample was placed in an aluminum pan and heated at a rate of 20 °C min^−1^ from room temperature to 250 °C under a N_2_ atmosphere (50 mL/min) after the sample was cooled quickly to −90 °C from the first scan. TEM images were recorded using a JEOL 2100 microscope (Japan) operated at 200 kV; ultrathin films were prepared using a Leica Ultracut microtome featuring a diamond knife and placed on a Cu grid coated with a carbon film. The P4VP-*b*-PS-DDSQ-PS-*b*-P4VP thin film was imaged after staining with I_2_ to display its P4VP segments; the PVPh-*b*-PS-DDSQ-PS-*b*-PVPh thin film was stained with RuO_4_ to display its PVPh segments.

## 3. Results

### 3.1. Synthesis of the Homopolymer PS-DDSQ-PS

**Figure 1:**
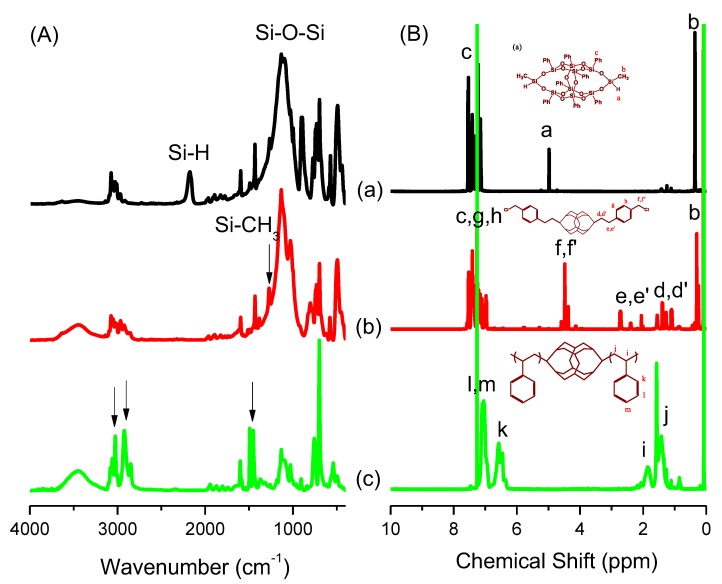
(**A**) FTIR and (**B**) ^1^H NMR spectra of (**a**) DDSQ, (**b**) VBC-DDSQ-VBC, and (**c**) PS-DDSQ-PS.

Scheme 1a–c displays the synthesis of the PS-DDSQ-PS homopolymer and the corresponding chemical structures of the DDSQ compounds, as confirmed using FTIR, ^1^H NMR spectroscopy and MALDI-TOF mass spectrometry. Figure 1 provides the FTIR and ^1^H NMR spectra of each compound obtained during the preparation of the PS-DDSQ-PS homopolymer. The FTIR spectrum of each DDSQ compound featured a strong signal for the Si–O–Si units at 1097 cm^−1^, with weak signals at 1261 cm^−1^ for the SiCH_3_ units of DDSQ [Figure 1A,a] and VBC-DDSQ-VBC [Figure 1A,b]. Furthermore, a signal at 2172 cm^−1^, representing Si–H stretching, appeared for pure DDSQ [Figure 1A,a], but it disappeared after the hydrosilylation of VBC with DDSQ [Figure 1A,b], indicating the success of the reaction. The ^1^H NMR spectrum of DDSQ [Figure 1B,a] featured signals for the aromatic protons at 7.50–7.14 ppm, the SiCH_3_ units at 0.36 ppm, and the SiH groups at 4.98 ppm. The signal for the SiH groups was absent from the spectrum of VBC-DDSQ-VBC [Figure 1B,b] after the vinyl units of VBC had undergone hydrosilylation. Signals for both the α- and β-configurations of the hydrosilylation product were evident; the ratio of β to α linkages in VBC-DDSQ-VBC was 1.22:1, based on integration of the signals e and e’ (or d and d’), again confirming the success of the reaction.

**Figure 2:**
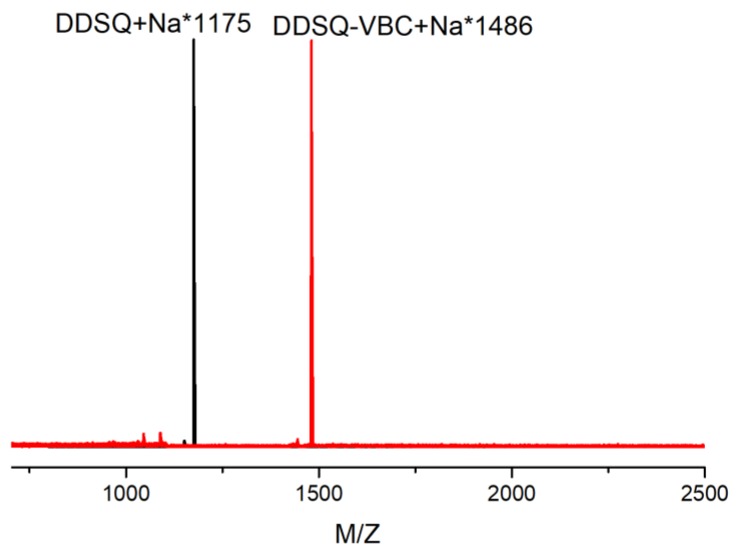
MALDI-TOF mass spectra of DDSQ and VBC-DDSQ-VBC.

Figure 2 displays the MALDI-TOF mass spectra of DDSQ and VBC-DDSQ-VBC, revealing their monodisperse mass distributions: [DDSQ + Na]^+^ at 1175 g/mol and [VBC-DDSQ-VBC + Na]^+^ at 1486 g/mol, consistent with their expected structures. Thus, the FTIR, NMR, and mass spectra confirmed the synthesis of the VBC-DDSQ-VBC initiator for ATRP.

**Figure 3:**
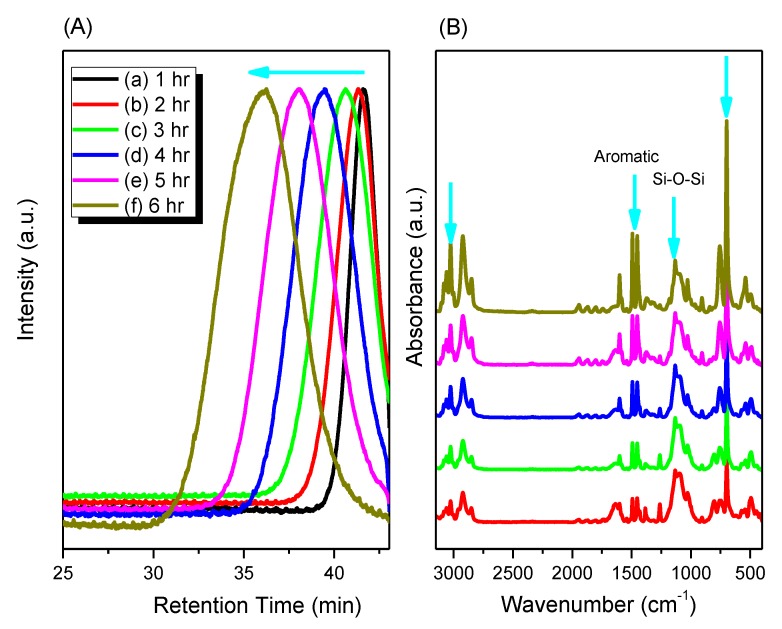
(**A**) SEC and (**B**) FTIR spectra of PS-DDSQ-PS homopolymers prepared with ATRP reaction times of (**a**) 1, (**b**) 2, (**c**) 3, (**d**) 4, (**e**) 5, and (**f**) 6 h.

Next, we synthesized PS-DDSQ-PS from the initiator VBC-DDSQ-VBC through ATRP [Scheme 1e]. We performed this ATRP with CuBr as the catalyst and PMEDTA as the ligand at 110 °C for 6 h; the conversion of the styrene monomer was 78% and the degree of polymerization (DP) was 112. Figure 1c presents the FTIR and ^1^H NMR spectra of the PS_56_-DDSQ-PS_56_ hybrid polymer; signals for the aromatic ring in the former appeared at 3030 and 1600 cm^−1^ [Figure 1A,c] while in the latter the aromatic protons appeared at 6.36–7.10 ppm and the main chain CH and CH_2_ protons appeared at 1.85 and 1.41 ppm [Figure 1B,c], indicating the successful ATRP yielding the PS-DDSQ-PS hybrid polymer. Figure 3A reveals shifts in the SEC distributions to lower retention times, indicating that the molecular weight increased upon increasing the reaction time, consistent with the FTIR spectra in Figure 3B of PS-DDSQ-PS hybrid polymers having PS segments of different molecular weights. Here, the intensity of the signal for Si–O–Si stretching of DDSQ was maintained, but the intensity of the corresponding absorptions of the PS segments at 3030, 1060, and 696 cm^−1^ all increased upon increasing the molecular weight of the PS segment [48].

**Figure 4:**
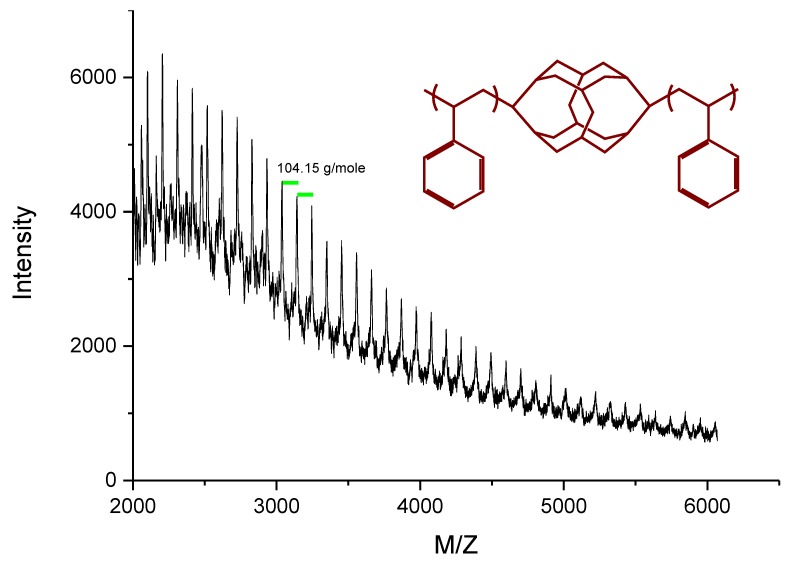
MALDI-TOF mass spectra of the hybrid polymer PS_20_-DDSQ-PS_20_.

Table 1 summarizes the molecular weights and PDIs of our PS-DDSQ-PS hybrid polymers. The well-defined structure of the PS_20_-DDSQ-PS_20_ hybrid polymer was confirmed from its MALDI-TOF mass spectrum (Figure 4), which presented only one apparent mass distribution. The mass difference between each pair of adjacent peaks was 104.15 g mol^−1^, equal to the expected mass of the repeat unit of a PS segment. Therefore, the NMR spectra, FTIR spectra, GPC traces, and mass spectra all confirmed the successful ATRP of the PS-DDSQ-PS hybrid polymers.

### 3.2. Synthesis of PtBuOS-b-PS-DDSQ-PS-b-PtBuOS and PVPh-b-PS-DDSQ-PS-b-PVPh

**Figure 5:**
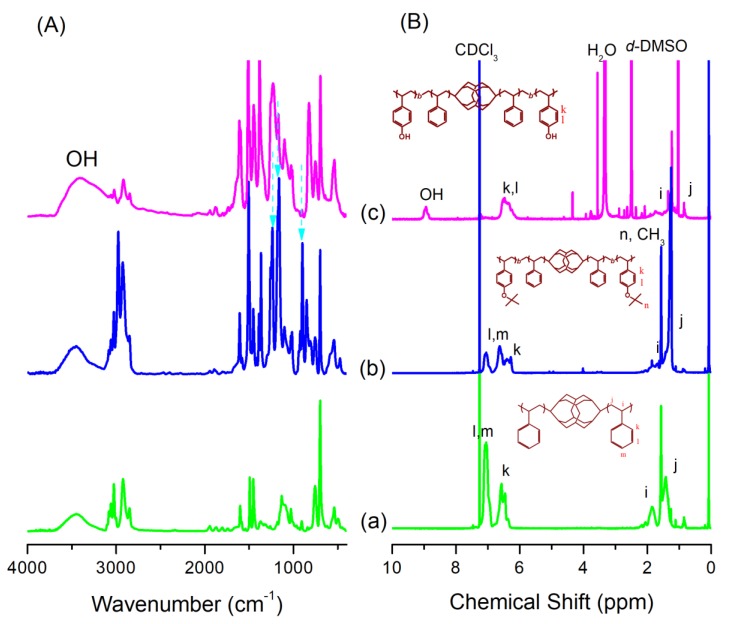
(**A**) FTIR and (**B**) ^1^H NMR spectra of (**a**) PS-DDSQ-PS, (**b**) PtBuOS-*b*-PS-DDSQ-PS-*b*-PtBuOS, and (**c**) PVPh-*b*-PS-DDSQ-PS-*b*-PVPh.

Scheme 1d–f summarizes the preparation of block copolymers based on DDSQ hybrids through ATRP. We used FTIR and ^1^H NMR spectra (Figure 5) to characterize the structures of the block copolymer hybrids PtBuOS-*b*-PS-DDSQ-PS-*b*- PtBuOS and PVPh-*b*-PS-DDSQ-PS-*b*-PVPh. Compared with the FTIR spectrum of the hybrid polymer PS_91_-DDSQ-PS_91_ [Figure 5A,a], that of the hybrid block copolymer PtBuOS-*b*-PS-DDSQ-PS-*b*-PtBuOS [Figure 5A,b] exhibited additional signals at 1243, 1163, and 905 cm^−1^ corresponding to the C–O and C–O–C stretching and C–H bending, respectively. Furthermore, the signal at 1.56 ppm in the ^1^H NMR spectrum of the hybrid block copolymer PtBuOS_78_-*b*-PS_91_-DDSQ-PS_91_-*b*-PtBuOS_78_ [Figure 5B,b], corresponding to the *tert*-butyl (C_4_H_9_) protons of the PtBuOS block segment, confirmed the successful ATRP of the tBuOS groups to give the PS-DDSQ-PS hybrid polymer. This signal at 1.56 ppm for the PtBuOS block segment essentially disappeared after treatment with N_2_H_4_, with a new peak appearing at 8.93 ppm representing the OH units of the PVPh block segment [Figure 5B,c],. Furthermore, the hydrolysis of the *tert*-butyl groups was evidenced by the signals at 1163 and 905 cm^−1^ decreasing in intensity, with a signal representing the presence of phenolic OH units for the PVPh block segment at 3400 cm^−^^1^, [Figure 5A,c],due to the self-association of hydrogen bonding units [42], suggesting the successful hydrolysis and the formation of the hybrid block copolymer PVPh-*b*-PS-DDSQ-PS-*b*-PVPh.

**Figure 6:**
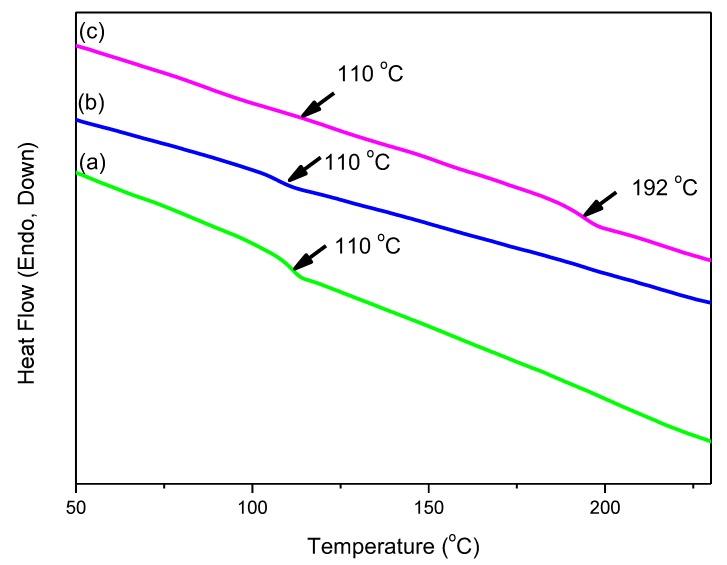
DSC thermograms of (**a**) PS-DDSQ-PS, (**b**) PtBuOS-*b*-PS-DDSQ-PS-*b*-PtBuOS, and (**c**) PVPh-*b*-PS-DDSQ-PS-*b*-PVPh.

**Figure 7:**
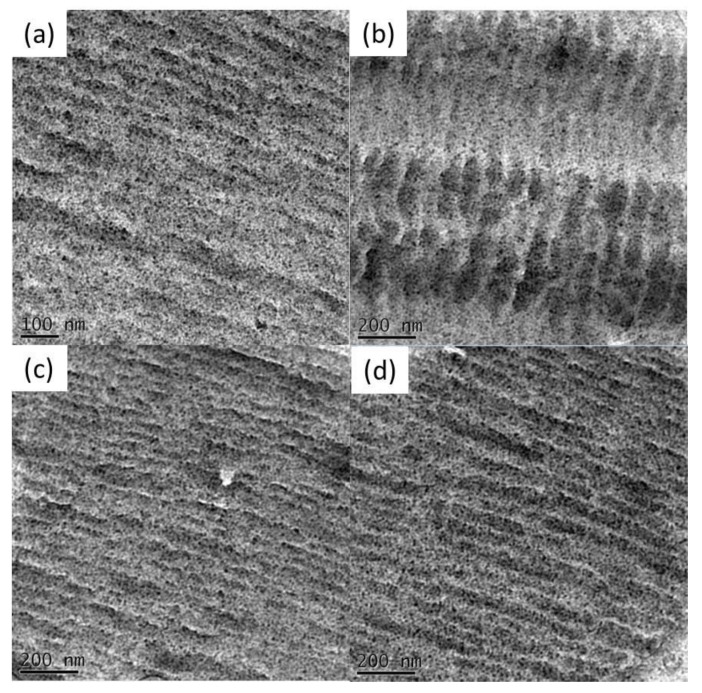
TEM images (**a**–**d**) of the hybrid block copolymer PVPh-*b*-PS-DDSQ-PS-*b*-PVPh.

Figure 6 displays the heating scans of the DSC analyses of the hybrid polymers PS-DDSQ-PS, PtBuOS-*b*-PS-DDSQ-PS-*b*-PtBuOS, and PVPh-*b*-PS-DDSQ-PS-*b*-PVPh. The glass transition temperature (*T*_g_) of the hybrid polymer PS-DDSQ-PS (110 °C) was higher than that of a PS homopolymer because the inorganic DDSQ nanoparticles enhanced the thermal properties [49]. For the block copolymer hybrid PtBuOS-*b*-PS-DDSQ-PS-*b*-PtBuOS, the value of *T*_g_ was also 110 °C, consistent with the value of *T*_g_ of a PtBuOS block segment being similar to that of a PS block segment [43]. Two values of *T*_g_ appeared, at 110 and 192 °C, for the block copolymer hybrid PVPh-*b*-PS-DDSQ-PS-*b*-PVPh, implying that microphase separation existed as a result of chemical incompatibility between the PS and PVPh block segments. The value of *T*_g_ of the PtBuOS block (110 °C) increased significantly to 192 °C for the PVPh segment in the DDSQ block copolymer because strong OH**···**OH hydrogen bonding decreases the free volume of the PVPh block segment. Figure 7 displays a TEM image of the block copolymer hybrid PVPh-*b*-PS-DDSQ-PS-*b*-PVPh; the apparent short-range order of a lamellae structure is similar to that we observed for the pure diblock copolymer PS-*b*-PVPh in a previous study. Furthermore, the dark regions in the TEM images may have arisen from aggregation of DDSQ nanoparticles having dimensions of approximately 2–3 nm, indicating that the DDSQ units had dispersed well in the block copolymer matrix.

### 3.3. Synthesis of P4VP-b-PS-DDSQ-PS-b-P4VP

**Figure 8:**
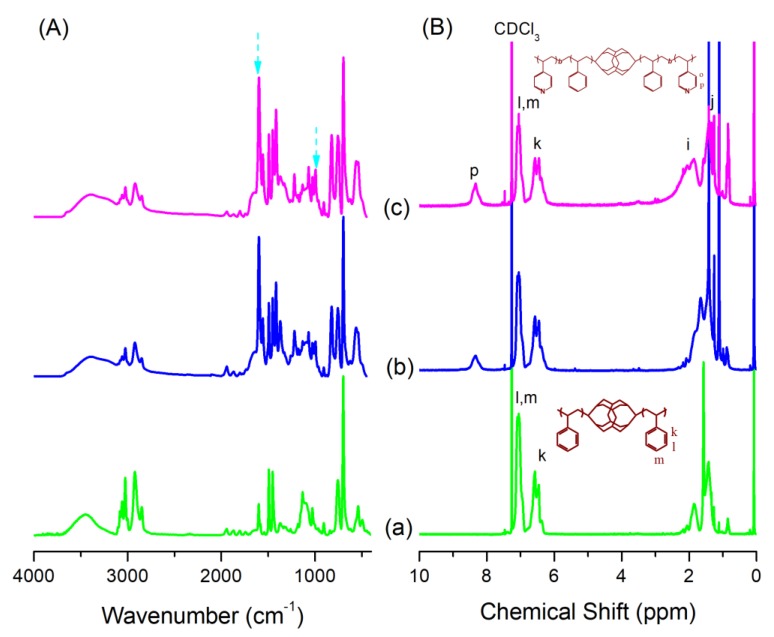
(**A**) FTIR and (**B**) ^1^H NMR spectra of (**a**) PS-DDSQ-PS, (**b**) P4VP_62_-*b*-PS_91_-DDSQ-PS_91_-*b*-P4VP_62_, and (**c**) P4VP_98_-*b*-PS_91_-DDSQ-PS_91_-*b*-P4VP_98_.

Scheme 1h displays the synthetic process toward the hybrid block copolymer P4VP-*b*-PS-DDSQ-PS-*b*-P4VP through sequential ATRP. Figure 8A presents the FTIR spectrum of the block copolymer P4VP-*b*-PS-DDSQ-PS-*b*-P4VP, revealing extra signals for pyridine stretching at 1590 and 993 cm^−1^, corresponding to the P4VP block segment, when compared with the spectrum of PS-DDSQ-PS. The intensities of these two characteristic signals for pyridine rings increased upon increasing the reaction time (8 and 12 h) of the 4-vinylpyridine monomer. Furthermore, Figure 8B displays typical ^1^H NMR spectra of the hybrid block copolymers PS-DDSQ-PS and P4VP-*b*-PS-DDSQ-PS-*b*-P4VP; Figure 8B,a,c) summarize the peak assignments. Signals representing the protons of the aromatic and pyridine rings appeared at 7.26–6.25 and 8.3 ppm, respectively; the integrated area for the signal of the extra pyridine units (peak n) at 8.3 ppm also increased upon increasing the reaction time (8 h and 12 h) of the 4-vinylpyridine monomer. We could use these ^1^H NMR spectra to calculate the molecular weights of the hybrid block copolymers P4VP_62_-*b*-PS_91_-DDSQ-PS_91_-*b*-P4VP_62_ (8 h) and P4VP_98_-*b*-PS_91_-DDSQ-PS_91_-*b*-P4VP_98_ (12 h).

**Figure 9:**
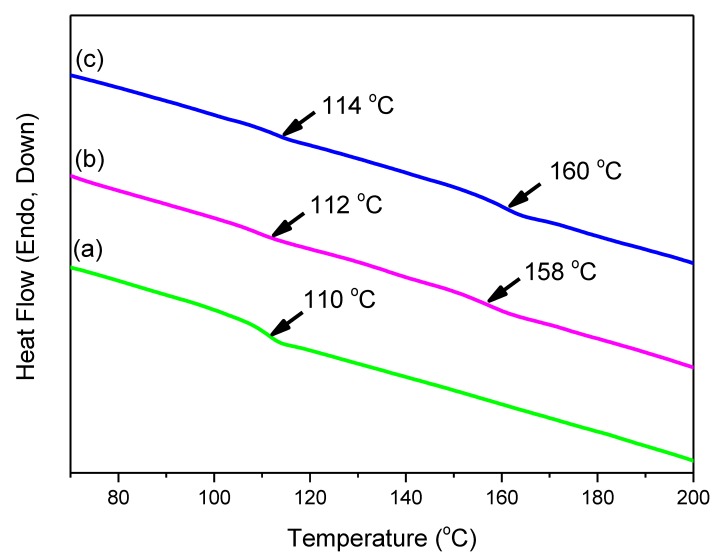
DSC thermograms of (**a**) PS-DDSQ-PS, (**b**) P4VP_62_-*b*-PS_91_-DDSQ-PS_91_-*b*-P4VP_62_, and (**c**) P4VP_98_-*b*-PS_91_-DDSQ-PS_91_-*b*-P4VP_98_.

Figure 9 presents the second heating scans of the DSC analyses of the hybrid block copolymers PS_91_-DDSQ-PS_91_, P4VP_62_-*b*-PS_91_-DDSQ-PS_91_-*b*-P4VP_62_, and P4VP_98_-*b*-PS_91_-DDSQ-PS_91_-*b*-P4VP_98_. Both of these P4VP-*b*-PS-DDSQ-PS-*b*-P4VP hybrid block copolymers displayed two glass transition temperatures, indicating that their two block segments were chemically incompatible, inducing microphase separation. The lower value of *T*_g_ represented the PS blocks, while the higher value of *T*_g_ represented the P4VP blocks. P4VP_62_-*b*-PS_91_-DDSQ-PS_91_-*b*-P4VP_62_ displayed values of *T*_g_ of 112 and 158 °C; for P4VP_98_-*b*-PS_91_-DDSQ-PS_91_-*b*-P4VP_98_ these values were 114 and 160 °C. Increasing the molecular weight of the P4VP block segment in the P4VP-*b*-PS-DDSQ-PS-*b*-P4VP hybrid block copolymer improved the thermal properties because of the greater hard confinement of this hybrid block copolymer.

**Figure 10:**
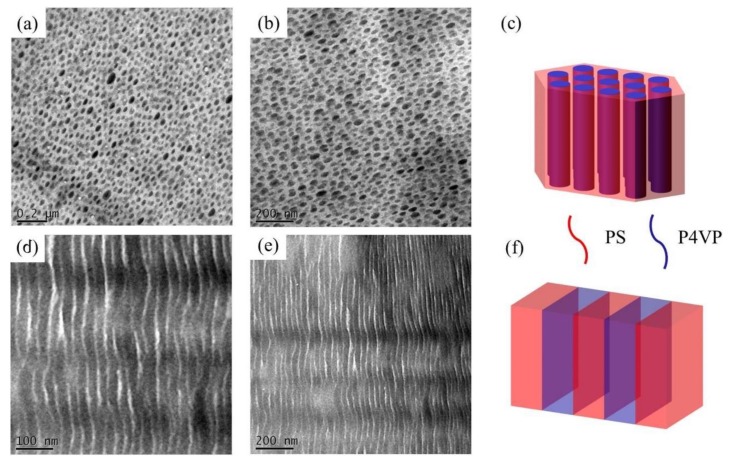
(**a**,**b**) TEM images of P4VP_62_-*b*-PS_91_-DDSQ-PS_91_-*b*-P4VP_62_. (**c**) Possible scheme for the corresponding cylinder structure. (**d**,**e**) TEM images of P4VP_98_-*b*-PS_91_-DDSQ-PS_91_-*b*-P4VP_98_. (**f**) Possible scheme for the corresponding lamellar structure.

Figure 10 displays TEM images of the hybrid block copolymers P4VP_62_-*b*-PS_91_-DDSQ-PS_91_-*b*-P4VP_62_ and P4VP_98_-*b*-PS_91_-DDSQ-PS_91_-*b*-P4VP_98_ stained with I_2_. The dark regions correspond to the P4VP block segments; the bright regions, the PS block segments. We observed a cylinder structure for the hybrid block copolymer P4VP_62_-*b*-PS_91_-DDSQ-PS_91_-*b*-P4VP_62_, as displayed in Figure 10a,b, with a minor phase (dark region) arising from the P4VP block segments, as displayed in Figure 10c, because the volume fraction of P4VP was approximately 31%. Increasing the molecular weight of the P4VP block segment, giving the hybrid block copolymer P4VP_98_-*b*-PS_91_-DDSQ-PS_91_-*b*-P4VP_98_, resulted in a lamellar structure, as displayed in Figure 10d,e; here, the volume fraction of P4VP block segments was approximately 52%, close to the 50% found in a typical lamellar structure [Figure 10f].

Thus, in this study we could indeed synthesize block copolymers (PVPh-*b*-PS-DDSQ-PS-*b*- PVPh, P4VP-*b*-PS-DDSQ-PS-*b*-P4VP) featuring strongly hydrogen bonding donor and acceptor block segments. In the near future we will investigate the hydrogen bonding interactions and self-assembly of PVPh-*b*-PS-DDSQ-PS-*b*-PVPh/P4VP-*b*-PS-DDSQ-PS-*b*-P4VP block copolymer mixtures.

## 4. Conclusions

We have synthesized two DDSQ-functionalized hydrogen bond donor and acceptor hybrid block copolymers—PVPh-*b*-PS-DDSQ-PS-*b*-PVPh and P4VP-*b*-PS-DDSQ-PS-*b*-P4VP—through sequential ATRP, and characterized them using FTIR spectroscopy, NMR spectroscopy, GPC, DSC, and MALDI-TOF mass spectrometry. Both the hybrid block copolymers PVPh-*b*-PS-DDSQ-PS-*b*-PVPh and P4VP-*b*-PS-DDSQ-PS-*b*-P4VP exhibited two glass transition temperatures, indicative of microphase separation with lamellar or cylindrical structures, which we confirmed through TEM image analyses. This facile approach allows the preparation, through controlled living radical polymerizations, of main chain–type block copolymers featuring DDSQ NPs at their junction point.

## Figures and Tables

**Table 1:** Molecular weights of PS-*b*-DDSQ-*b*-PS polymers used in this study.

Sample	PS (*M*_n_) ^a^	PDI ^b^
1 h	4000	1.05
2 h	4300	1.16
3 h	5400	1.22
4 h	7600	1.27
5 h	11,100	1.31
6 h	19,000	1.42

^a^: Determined from ^1^H NMR spectra. ^b^: Determined through GPC analysis.
